# An external validation of the nocera nomogram: Predicting non-organ confined stage of ≥pT3 in cT1 clear cell renal cell carcinoma

**DOI:** 10.3389/fonc.2022.1019057

**Published:** 2022-10-10

**Authors:** Mike Wenzel, Benedikt Hoeh, Jessica Rührup, Hanna Gambetta, Luigi Nocera, Christoph Würnschimmel, Zhe Tian, Pierre I. Karakiewicz, Alberto Briganti, Felix K.H. Chun, Frederik C. Roos, Andreas Becker, Marieke J. Krimphove

**Affiliations:** ^1^ Department of Urology, University Hospital Frankfurt, Goethe University Frankfurt am Main, Frankfurt, Germany; ^2^ Cancer Prognostics and Health Outcomes Unit, Division of Urology, University of Montreal Health Center, Montreal, QC, Canada; ^3^ Division of Experimental Oncology/Unit of Urology, URI, Urological Research Institute, IRCCS San Raffaele Scientific Institute, Milan, Italy; ^4^ Department of Urology, Luzerner Kantonspital, Lucerne, Switzerland; ^5^ Department of Health Science and Medicine, Univerity of Lucerne, Lucerne, Switzerland

**Keywords:** clear cell, kidney cancer, renal cell carcinoma, external validation, nomogram

## Abstract

**Background:**

Only one previously published study by Nocera et al. addressed the risk of upstaging to ≥pT3 in cT1 clear cell renal cell carcinoma (ccRCC) by using characteristics of the R.E.N.A.L and PADUA score (age, tumor size, rim location, exophytic rate, polar involvement) developing an accurate nomogram. However, this nomogram has never been externally validated yet.

**Material and methods:**

The study cohort consisted of 288 patients with cT1a-b ccRCC, diagnosed between 2008-2021 at the University Hospital Frankfurt, Germany. Analyses addressed clinical, tumor and radiographic characteristics. The external validation of the nomogram relied on accuracy calculations derived from the area under the curve of the receiver operator characteristic analysis.

**Results:**

Overall, 11.8% (n=34) patients harbored ≥pT3 ccRCC. Median radiographic tumor size (3.6 *vs*. 5.3cm), R.E.N.A.L. (8 *vs*. 9 points) and PADUA score (9 *vs*. 11 points), as well as proportions of renal sinus involvement (82.4% *vs*. 51.6%), renal hilus involvement (44.1 *vs*. 13.0%), and medial rim location significantly differed between the pT1-2 and ≥pT3 group (all p ≤ 0.01). In subgroup analyses of small renal mass ccRCC patients (<4cm, cT1a), only 3.8% (n=6) patients had ≥pT3 pathology. Upstaged patients were significantly older and more frequently had endophytic tumor than pT1-2 counterparts (p<0.05). The external validation of the Nocera nomogram showed a good accuracy of 76.6%. Using the suggested cut-off of 21%, 26.5% of patients exhibited ≥pT3 ccRCC. Conversely, within patients below cut-off, 5.9% patients exhibited ≥pT3 ccRCC.

**Conclusion:**

We reported the first external validation of the nomogram addressing the risk of ≥pT3 in cT1 ccRCC patients, demonstrating a good accuracy, with a low false-negative rate. Therefore, the nomogram can accurately be used for patients’ counselling and treatment decision making.

## Introduction

There are several treatment options available for cT1 clear cell renal cell carcinoma (ccRCC) according to EAU and NCCN guidelines (1, 2). However, an undeniable proportion of 4-11% of cT1 ccRCC patients harbor non-organ defined ≥pT3 disease at final pathology, which is associated with limited cancer-specific survival (3–7).

Due to health care restrictions since the onset of COVID-19 pandemic, non-life-threatening surgeries have been and are still being postponed in multiple cases. It is therefore crucial to identify ccRCC patients with high risk of harboring unfavorable pathological stage for rapid surgical treatment (8). Nowadays, among others, radiographic kidney parameters are frequently used to predict complexity and periprocedural outcomes of the surgical treatment of RCC, as used for the R.E.N.A.L. or PADUA score (9–13). Only one multi-institutional study group developed a predicting model and nomogram using these easily applicable radiographic parameters to predict the risk of upstaging to ≥pT3 stage in patients surgically treated for cT1 ccRCC (6). This nomogram by Nocera et al. reaches an accuracy of 81%, however, since databases with sufficient sample size of ≥pT3 ccRCC are scant, the Nocera nomogram has never been externally validated in a different patient cohort. Moreover, the study by Nocera et al. did not distinguish between cT1a (<4cm tumor size) *vs*. cT1b (4-7cm tumor size) patients. However, this distinction is clinically important as cT1a small renal masses (SRM) patients may undergo active surveillance as feasible treatment option (1, 14, 15).

We addressed this void and relied on our institutional ccRCC surgical database to externally validate the accuracy of the Nocera nomogram, predicting ≥pT3 in cT1 ccRCC patients. Moreover, we investigated characteristics of SRM patients with ≥pT3 pathological stage.

## Material and methods

### Study population

After approval of the local ethic committee, all patients with ccRCC histology and surgical treatment (partial nephrectomy or nephrectomy) at the Department of Urology, University Hospital Frankfurt, Germany, were retrospectively identified. The patients’ inclusion duration was between 01/2008 and 12/2021. Exclusion criteria consisted of patients with other histology than ccRCC, patients younger than 18 years or older than 80 years, as well as patients with multiple radiographic tumor lesions or metastatic or nodal positive disease, according to the inclusion criteria of the Nocera nomogram (6). These criteria yielded 288 ccRCC patients.

### Covariates and endpoints

Covariates of the nomogram validation consisted of age at diagnosis (continuously), tumor size in cm (continuously), rim location (medial *vs*. lateral), exophytic rate (<50% *vs*. ≥50% *vs*. endophytic), polar involvement (yes *vs*. no), as previously reported and defined (6, 9, 10). Primary endpoint of the current study was the prediction of ≥pT3 ccRCC at final pathological stage.

### Statistical analysis

Descriptive statistics included frequencies and proportions for categorical variables. Medians and interquartile ranges (IQR) were reported for continuous variables. Statistically significant differences between groups were identified using the Chi-square test for categorical and t-test and Kruskal-Wallis test for continuous variables.

External validation of the Nocera nomogram derived from the initial odds ratios (OR) of the above-mentioned covariates of the study by Nocera et al. (6). Calibration plots depicted the relationship between prediction by Nocera nomogram *vs*. actual probability of ≥pT3 in cT1 ccRCC patients and resulted in a ROC-derived area under the curve (AUC) accuracy. Moreover, decision curve analyses (DCA) tested the net-benefit related to the use of the Nocera nomogram (16). Finally, systematic analyses of several possible model probability cut-offs were performed. All tests were two sided with a level of significance set at p<0.05. R software environment for statistical computing and graphics (version 3.4.3) was used for all analyses.

## Results

### Descriptive and radiographic characteristics of the validation cohort

Overall, 288 cT1 ccRCC patients were identified and included in the current study (**Table 1**). Median age was 65 years (IQR 56-72) and most patients were male (71.2%). Of all cT1 ccRCC patients, 54.5% (n=157) harbored radiographically cT1a stage *vs*. 45.5% (n=131) cT1b stage. Median R.E.N.A.L. score was 8 (IQR 7-9) and PADUA score 10 (IQR 8-11). High surgical complexity according to R.E.N.A.L. and PADUA score was observed in 22.6 (n=65) and 50.3% (n=145) of cT1 ccRCC patients, respectively. Detailed R.E.N.A.L. and PADUA score information are summarized in **Table 1**.

**Table 1:** Descriptive characteristics of 288 clear cell renal cell carcinoma patients, surgically treated between 2008-2021 at the University Hospital Frankfurt, stratified according to pT1-2 vs. ≥pT3 stage.

Variable		Overall n=288	pT1-2 n=254 (88.2%)	≥pT3 n=34 (11.8%)	P value
**Age**	Median (IQR)	65 (56-72)	64 (55-71)	67 (61-73)	0.11
**Tumor size, cm**	Median (IQR)	3.9 (2.7-5.1)	3.6 (2.6-4.8)	5.3 (4.4-5.7)	<0.001
**Sex**	male	207 (71.9)	182 (71.7)	25 (73.5)	1
	female	81 (28.1)	72 (28.3)	9 (26.5)	
**cT**	cT1a	157 (54.5)	151 (59.4)	6 (17.6)	<0.001
	cT1b	131 (45.5)	103 (40.6)	28 (82.4)	
**Surgical approach**	PN	242 (84.0)	227 (89.4)	15 (44.1)	<0.001
	RN	46 (16.0)	27 (10.6)	19 (55.9)	
**Sinus involvement**	Involved	159 (55.2)	131 (51.6)	28 (82.4)	0.001
	Not involved	129 (44.8)	123 (48.4)	6 (17.6)	
**Exophytic rate**	≥50%	67 (23.3)	57 (22.4)	10 (29.4)	0.5
	<50%	180 (62.5)	162 (63.8)	18 (52.9)	
	Endophytic	41 (14.2)	35 (13.8)	6 (17.6)	
**Location relative to polar line**	>50% crossing	142 (49.3)	120 (47.2)	22 (64.7)	0.15
	<50% crossing	82 (28.5)	76 (29.9)	6 (17.6)	
	Not crossing	64 (22.2)	58 (22.8)	6 (17.6)	
**Side**	Anterior	119 (41.3)	107 (42.1)	12 (35.3)	0.7
	Posterior	126 (43.8)	109 (42.9)	17 (50.0)	
	Unclear	43 (14.9)	38 (15.0)	5 (14.7)	
**Hilus involvement**	involved	48 (16.7)	33 (13.0)	15 (44.1)	<0.001
	not involved	240 (83.3)	221 (87.0)	19 (55.9)	
**Rim location**	Lateral	167 (58.0)	155 (61.0)	12 (35.3)	<0.01
	Medial	121 (42.0)	99 (39.0)	22 (64.7)	
**R.E.N.A.L. score**	Median (IQR)	8 (7-9)	8 (7-9)	9 (9-10)	<0.001
**R.E.N.A.L. score**	Low	41 (14.2)	40 (15.7)	1 (2.9)	<0.01
	Intermediate	182 (63.2)	164 (64.6)	18 (52.9)	
	High	65 (22.6)	50 (19.7)	15 (44.1)	
**Padua score**	Median (IQR)	10 (8-11)	9 (8-11)	11 (10-12)	<0.001
**Padua groups**	Low	54 (18.8)	52 (20.5)	2 (5.9)	<0.001
	Intermediate	89 (30.9)	86 (33.9)	3 (8.8)	
	High	145 (50.3)	116 (45.7)	29 (85.3)	

IQR, Interquartile range; PN, Partial nephrectomy; RN, Radical nephrectomy.

### Radiographic characteristics of pT1-2 *vs*. ≥pT3 ccRCC patients

Of all 288 cT1 ccRCC patients, 88.2% (n=254) harbored pT1-2 final pathological stage (**Table 1**). Conversely, 11.8% (n=34) exhibited ≥pT3 stage. In direct comparison, ≥pT3 patients had greater median tumor size (5.3 *vs*. 3.6 cm) and higher proportions of cT1b stage (82.4% *vs*. 40.6%, both p<0.001). Moreover, higher radiographic proportions of renal sinus involvement (82.4% *vs*. 51.6%), renal hilus involvement (44.1 *vs*. 13.0%) and medial rim location (64.7 *vs*. 39.0%) were observed (all p<0.01) in ≥pT3 patients. Conversely, rates of endophytic (17.6% *vs*. 13.8%) and posterior tumor side (50.0% *vs*. 42.9%) did not differ between groups (both p≥0.5). Finally, median R.E.N.A.L. (9 *vs*. 8) and PADUA score (11 *vs*. 9) were significantly higher in ≥pT3 patients *vs*. pT1-2 patients.

In subgroup analyses of SRM cT1a ccRCC patients (n=157), 3.8% (n=6) patients harbored ≥pT3 ccRCC (**Table 2**). Of those, ≥pT3 patients were significantly older (71 *vs*. 63 years) and had significantly higher rates of completely endophytic tumors (50.0 *vs*. 17.9%, both p ≤ 0.03) relative to pT1-2 ccRCC patients. However, median R.E.N.A.L. (8 *vs*.8) and PADUA score (9 *vs*. 8) did not differ between ≥pT3 patients *vs*. pT1-2 ccRCC patients (both p>0.05).

**Table 2:** Descriptive characteristics of 157 small renal mass (SRM, cT1a) clear cell renal cell carcinoma patients, surgically treated between 2008-2021 at the University Hospital Frankfurt, stratified according to pT1-2 vs. ≥pT3 stage.

Variable		Overall n=157	pT1-2 n=151 (96.2%)	≥pT3 n=6 (3.8%)	P value
**Age**	Median (IQR)	64 (54-71)	63 (54-71)	71 (69-74)	0.03
**Tumor size, cm**	Median (IQR)	2.8 (2.3-3.4)	2.8 (2.3-3.4)	3.2 (2.4-3.6)	0.4
**Sex**	male	114 (72.6)	108 (71.5)	6 (100)	0.3
	female	43 (27.4)	43 (28.5)	0 (0)	
**Surgical approach**	PN	152 (96.8)	148 (98.0)	4 (66.7)	<0.01
	RN	5 (3.2)	3 (2.0)	2 (33.3)	
**Sinus involvement**	Involved	58 (36.9)	55 (36.4)	3 (50.0)	0.8
	Not involved	99 (63.1)	96 (63.6)	3 (50.0)	
**Exophytic rate**	≥50%	21 (13.4)	19 (12.6)	2 (33.3)	0.025
	<50%	106 (67.5)	105 (69.5)	1 (16.7)	
	Endophytic	30 (19.1)	27 (17.9)	3 (50.0)	
**Location relative to polar line**	>50% crossing	66 (42)	64 (42.4)	2 (33.3)	0.4
	<50% crossing	48 (30.6)	47 (31.1)	1 (16.7)	
	Not crossing	43 (27.4)	40 (26.5)	3 (50.0)	
**Side**	Anterior	74 (47.1)	70 (46.4)	4 (66.7)	0.5
	Posterior	68 (43.3)	66 (43.7)	2 (33.3)	
	Unclear	15 (9.6)	15 (9.9)	0 (0)	
**Hilus involvement**	involved	10 (6.4)	10 (6.6)	0 (0)	1
	not involved	147 (93.6)	141 (93.4)	6 (100)	
**Rim location**	Lateral	101 (64.3)	97 (64.2)	4 (66.7)	1
	Medial	56 (35.7)	54 (35.8)	2 (33.3)	
**R.E.N.A.L. score**	Median (IQR)	8 (7-9)	8 (7-9)	8 (7-9)	0.8
**R.E.N.A.L. score**	Low	37 (23.6)	36 (23.8)	1 (16.7)	0.8
	Intermediate	106 (67.5)	102 (67.5)	4 (66.7)	
	High	14 (8.9)	13 (8.6)	1 (16.7)	
**Padua score**	Median (IQR)	8 (7-10)	8 (7-10)	9 (7-11)	0.7
**Padua groups**	Low	50 (31.8)	48 (31.8)	2 (33.3)	0.4
	Intermediate	60 (38.2)	59 (39.1)	1 (16.7)	
	High	47 (29.9)	44 (29.1)	3 (50.0)	

IQR, Interquartile range; PN, Partial nephrectomy; RN, Radical nephrectomy.

### External validation of the Nocera nomogram

**Figure 1:**
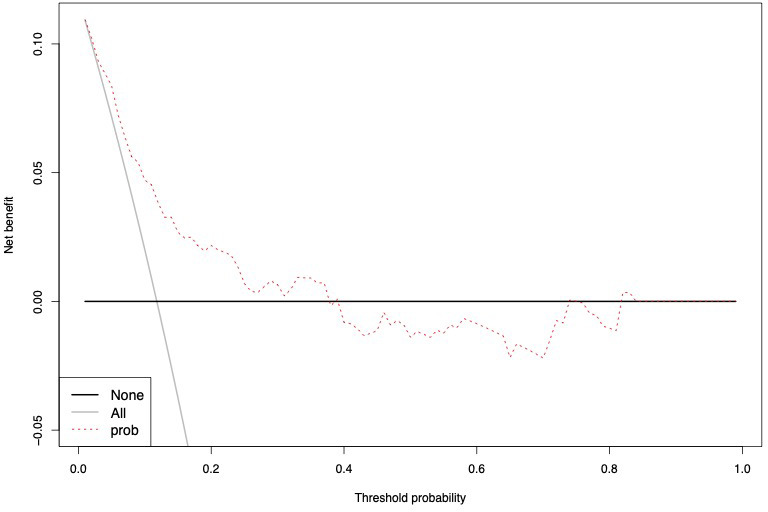
Calibration curve depicting the relationship between prediction by Nocera nomogram *vs* actual probability of ≥pT3 in cT1 ccRCC patients, resulting in a ROC-derived area under the curve (AUC) accuracy.

**Figure 2:**
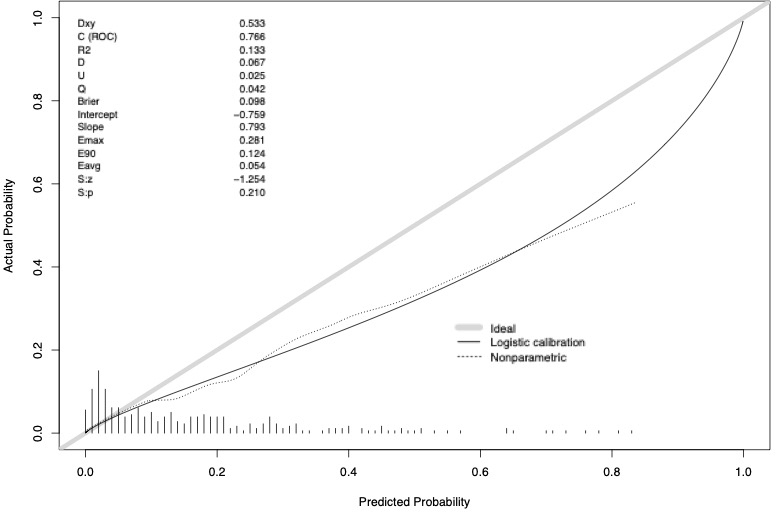
Decision curve analysis depicting the net benefit of the Nocera nomogram in the external validation cohort (dotted red line), relative to treat none (black line) or treat all (gray line). The nomogram shows a greater net benefit until a threshold probability of 0.4.

The accuracy of the external validation of the Nocera nomogram resulted in 76.6% of ≥pT3 prediction, relying on the clinical and radiographic variables of age at diagnosis, tumor size, rim location, exophytic rate and polar involvement. The relationship between predicted probability by the Nocera nomogram of ≥pT3 ccRCC and observed of ≥pT3 ccRCC rates is depicted in the calibration plot (**Figure 1**). Here, the nomogram predicted values overestimated the risk of ≥pT3 ccRCC at values >10%. DCA graphically depicted the net benefit of the nomogram in the external validation cohort (**Figure 2**). The use of the nomogram resulted in greater net benefit, at threshold probabilities >0.4, relative to both competing strategies (treat non/treat all).

Finally, we tabulated various nomogram cut-offs for prediction of ≥pT3 ccRCC (**Table 3**), according to number and percentage of correctly identified patients (true positive) *vs*. those that were incorrectly classified (false positive).

**Table 3:** Analyses of nomogram cutoffs in 288 cT1 clear cell-renal cell carcinoma patients.

Cutoff (%)	Patients above nomogram cutoff (%)	Number of ≥pT3 patients above cutoff [PPV] (%)(true positive)	Patients below nomogram cutoff (%)	Number of ≥pT3 patients below cutoff [1-NPV] (%)(false negative)	Specificity
**1**	278 (96.5)	34 (12.2)	10 (3.5)	0 (0.0)	3.9
**2**	259 (89.9)	34 (13.1)	29 (10.1)	0 (0.0)	11.4
**3**	232 (80.6)	33 (14.2)	56 (19.4)	1 (1.8)	21.7
**4**	213 (74.0)	33 (15.5)	75 (26.0)	1 (1.3)	29.1
**5**	202 (70.1)	33 (16.3)	86 (29.9)	1 (1.2)	33.5
**6**	191 (66.3)	31 (16.2)	97 (33.7)	3 (3.1)	37.0
**7**	184 (63.9)	30 (16.3)	104 (36.1)	4 (3.8)	39.4
**8**	176 (61.1)	29 (16.5)	112 (38.9)	5 (4.5)	42.1
**9**	165 (57.3)	29 (17.6)	123 (42.7)	5 (4.1)	46.5
**10**	158 (54.9)	28 (17.7)	130 (45.1)	6 (4.6)	48.8
**11**	149 (51.7)	28 (18.8)	139 (48.3)	6 (4.3)	52.4
**12**	144 (50.0)	27 (18.8)	144 (50.0)	7 (4.9)	53.9
**13**	137 (47.6)	26 (19.0)	151 (52.4)	8 (5.3)	56.3
**14**	128 (44.4)	26 (20.3)	160 (55.6)	8 (5.0)	59.8
**15**	123 (42.7)	25 (20.3)	165 (57.3)	9 (5.5)	61.4
**16**	119 (41.3)	25 (21.0)	169 (58.7)	9 (5.3)	63.0
**17**	112 (38.9)	25 (22.3)	176 (61.1)	9 (5.1)	65.7
**18**	105 (36.5)	24 (22.9)	183 (63.5)	10 (5.5)	68.1
**19**	97 (33.7)	23 (23.7)	191 (66.3)	11 (5.8)	70.9
**20**	90 (31.2)	23 (25.6)	198 (68.8)	11 (5.6)	73.6
**21**	83 (28.8)	22 (26.5)	205 (71.2)	12 (5.9)	76.0
**22**	76 (26.4)	21 (27.6)	212 (73.6)	13 (6.1)	78.3
**23**	74 (25.7)	21 (28.4)	214 (74.3)	13 (6.1)	79.1
**24**	71 (24.7)	20 (28.2)	217 (75.3)	14 (6.5)	79.9
**25**	70 (24.3)	19 (27.1)	218 (75.7)	15 (6.9)	79.9
**26**	66 (22.9)	18 (27.3)	222 (77.1)	16 (7.2)	81.1
**27**	64 (22.2)	18 (28.1)	224 (77.8)	16 (7.1)	81.9
**28**	60 (20.8)	18 (30.0)	228 (79.2)	16 (7.0)	83.5
**29**	53 (18.4)	17 (32.1)	235 (81.6)	17 (7.2)	85.8
**30**	49 (17.0)	16 (32.7)	239 (83.0)	18 (7.5)	87.0
**31**	47 (16.3)	15 (31.9)	241 (83.7)	19 (7.9)	87.4
**32**	44 (15.3)	15 (34.1)	244 (84.7)	19 (7.8)	88.6
**33**	40 (13.9)	15 (37.5)	248 (86.1)	19 (7.7)	90.2
**34**	39 (13.5)	15 (38.5)	249 (86.5)	19 (7.6)	90.6
**35**	38 (13.2)	15 (39.5)	250 (86.8)	19 (7.6)	90.9
**36**	38 (13.2)	15 (39.5)	250 (86.8)	19 (7.6)	90.9
**37**	37 (12.8)	15 (40.5)	251 (87.2)	19 (7.6)	91.3
**38**	35 (12.2)	13 (37.1)	253 (87.8)	21 (8.3)	91.3
**39**	33 (11.5)	13 (39.4)	255 (88.5)	21 (8.2)	92.1
**40**	31 (10.8)	11 (35.5)	257 (89.2)	23 (8.9)	92.1

Predicted probabilities ranged from 1 to 80% (**Table 3**). The use of a suggested probability cut-off of 21% by Nocera et al. identified 83 patients (28.8%) that were situated above that cut-off, indicating an elevated risk of ≥pT3 ccRCC. Within those patients, 28 patients (26.5%) exhibited ≥pT3 ccRCC at final pathology. Conversely, within 205 (71.2%) of cT1 patients that were placed below the probability cut-off of 21%, 12 (5.9%) patients exhibited ≥pT3 ccRCC after surgery.

The use of a less strict cut-off of 14% resulted in 128 (44.4%) patients that were placed above that cut-off, indicative of an elevated risk of ≥pT3 in the external validation cohort. Within these patients, 26 (20.3%) harbored ≥pT3 ccRCC at final pathology. Conversely, within 160 cT1 (55.6%) patients that were placed below the probability cut-off of 14%, 8 (5.0%) patients harbored ≥pT3 ccRCC.

## Discussion

Predicting the risk of presence of ≥pT3 ccRCC may substantially affect treatment decision making in cT1 ccRCC patients. Today, little is known about the rate of upstaging from cT1 to ≥pT3 ccRCC disease based on clinical and radiographic characteristics. Moreover, it is unknown whether such upgrading may be accurately predicted in another patient cohort than the one underlying the nomogram developed by Nocera et al. (6). Finally, the chance of upstaging to ≥pT3 ccRCC in SRM patients has never been formally addressed. This study was designed to address these knowledge gaps and lead to the following noteworthy findings.

First, we observed that upstaging to ≥pT3 ccRCC affects as many as 11.8% of all cT1 ccRCC patients treated with either partial or radical nephrectomy. Compared to patients with pT1-2 ccRCC final pathology, most radiographic parameters as used in R.E.N.A.L. and PADUA score systems showed adverse features in patients upstaged to ≥pT3 ccRCC in final pathology. This finding underscores the original use of radiographic parameters as predictors for surgical complexity and risk factors for pathological upstaging and is in line with current literature. For example, a recently published study predicting pT3a ccRCC in clinically cT1-2 patients also found a greater median tumor size and a higher median R.E.N.A.L score in pT3a ccRCC patients, relative to pT1-2 patients (17). However, this study did not exclusively examine cT1 tumors but also included approximately 21% of cT2 kidney cancer patients, which explain the rate of 19% pT3a at final pathology as they are known to harbor a higher risk of upstaging (18–20). Two other studies by Mouracade et al. and Ramaswamy et al. found a rate of 11.8% and 13.3% of upstaging to pT3a in cT1 ccRCC patients, respectively. These studies emphasize that radiographically complex tumors are at higher risk of upstaging (21, 22).

Second, the external validation of the Nocera nomogram resulted in a 76.6% (n=288, ≥pT3 ccRCC rate of 11.8%) compared to the initial nomogram accuracy of 81.0% (n=236, pT3a ccRCC rate of 10.6%). This external validated accuracy exceeds even those of newly developed nomograms (23). Therefore, the external validation of the Nocera nomogram within our larger population from another country is clinically very important as it confirms that the nomogram is robust and may be generalizable. Moreover, it indicates that easily applicable clinical and radiographic parameters can accurately be used to predict the risk of upstaging to ≥pT3 ccRCC. Specifically, the initially suggested threshold of 21% by Nocera et al. seems adequate as it identifies 28.8% of patients at risk of ≥pT3 ccRCC at final pathology with 26.5% of those actually harboring ≥pT3 ccRCC. Conversely, 71.2% are at low risk of upstaging as confirmed in a low percentage of only 5.9% being upstaged. The numbers are comparable to those in Nocera’s study. With that said, setting a cut-off at 21% will result in every fourth to third patient above the cut-off harboring upstaging to ≥pT3 ccRCC, while the false-negative rate is extreme low with constantly <6% in both studies. Finally, the miscalibration at predicted probabilities >20% in the current study are outside the clinical importance.

Third, we found that only 3.8% of SRM (cT1a, <4cm) patients harbored ≥pT3 ccRCC at final pathology. Moreover, we observed that SRM patients with ≥pT3 ccRCC at final pathology were older and harbored more frequently endophytic tumors than their pT1 counterparts with SRM. These observations are also consistent with the current literature. For example, studies by Ball et al. and Srivastava et al. also found 3.8% and 4.2% of SRM patients with upstaging to pT3a ccRCC at final pathology, respectively (19, 24). These observations are particularly important because SRMs are often treated with active surveillance as their annual growth rate is extremely low (25). Our findings support this approach since only approximately one out of thirty SRM patients will harbor locally advanced ccRCC after surgery (26). Moreover, these observations may indicate that these patients may not need immediate surgical therapy and can be delayed in times with restricted health care capacity as evoked during the COVID-19 pandemic (8, 27–29). Unfortunately, due to sample size limitations, we were not able to do further analyses such as logistic regression models or improvement of the Nocera nomogram with a subgroup of cT1a kidney tumors only.

In addition, our study is not devoid further limitations beginning with the retrospective character of the study. Moreover, no central pathological or radiological review was given. Both, Nocera’s and our study were based on European cohorts limiting the generalizability of the findings to other populations, since they may harbor different outcomes (30). Further studies in other populations are needed to test its generalizability. Finally, since tumor biology and oncological outcomes of non-ccRCC differ, findings are only usable for ccRCC patients (3, 31). Further investigations for non-ccRCC patients are needed in this context.

Taken together, we observed an overall risk of 11.8% and 3.6% of upstaging to ≥pT3 ccRCC in all cT1 and SRM (cT1a) patients, respectively. The external validation of the Nocera nomogram yielded a good accuracy. Moreover, cut-offs between 14-21% indicate a low false-negative rate after applying the nomogram. In summary, the current Nocera nomogram can be used by clinicians for treatment decision making, especially when delayed surgery may be considered.

## Data availability statement

The raw data supporting the conclusions of this article will be made available by the authors, without undue reservation.

## Ethics statement

This study was reviewed and approved by UCT Frankfurt. Written informed consent for participation was not required for this study in accordance with the national legislation and the institutional requirements.

## Author contributions

Conceptualization: MW, BH, AnB, MK. Data curation: MW, JR, HG, FCR, AnB, MK. Formal analysis: MW, BH, ZT, LN. Funding acquisition: — Investigation: MW, BH, LN, CW. Methodology: MW, ZT Supervision: PK, AlB, FC. Validation: PK, AlB, FC. All authors contributed to the article and approved the submitted version.

## Conflict of interest

The authors declare that the research was conducted in the absence of any commercial or financial relationships that could be construed as a potential conflict of interest.

## Publisher’s note

All claims expressed in this article are solely those of the authors and do not necessarily represent those of their affiliated organizations, or those of the publisher, the editors and the reviewers. Any product that may be evaluated in this article, or claim that may be made by its manufacturer, is not guaranteed or endorsed by the publisher.
